# Lecanemab: Appropriate Use Recommendations

**DOI:** 10.14283/jpad.2023.30

**Published:** 2023

**Authors:** J. Cummings, L. Apostolova, G.D. Rabinovici, A. Atri, P. Aisen, S. Greenberg, S. Hendrix, D. Selkoe, M. Weiner, R.C. Petersen, S. Salloway, Alzheimer’s Disease and Related Disorders Therapeutics Work Group

**Affiliations:** 1 Chambers-Grundy Center for Transformative Neuroscience, Department of Brain Health, School of Integrated Health Sciences, University of Nevada Las Vegas (UNLV), Las Vegas, NV; USA; 2 Departments of Neurology, Radiology, Medical and Molecular Genetics, Indiana University School of Medicine, Indianapolis, Indiana, USA; 3 Memory and Aging Center, Department of Neurology, Weill Institute for Neurosciences and Department of Radiology and Biomedical Imaging; University of California, San Francisco, San Francisco, CA, USA; 4 Banner Sun Health Research Institute, Banner Health, Sun City, AZ; Center for Brain/Mind Medicine, Harvard Medical School, Boston, MA, USA; 5 Alzheimer’s Treatment Research Institute, University of Southern California, San Diego, CA, USA; 6 Deparment of Neurology, Massachusetts General Hospital, Harvard Medical School, Boston, MA, USA; 7 Pentara Corporation, Millcreek Utah, USA; 8 Ann Romney Center for Neurologic Diseases, Department of Neurology, Brigham and Women’s Hospital, Harvard Medical School, Boston, Massachusetts, USA; 9 Departments of Radiology and Biomedical Imaging, Medicine, Psychiatry and Neurology, University of California San Francisco, San Francisco, CA, USA; 10 Department of Neurology, Mayo Clinic, Rochester, MN, USA; 11 Butler Hospital and Warren Alpert Medical School of Brown University, Providence RI, USA

**Keywords:** Alzheimer’s disease, lecanemab, aducanumab, donanemab, appropriate use recommendations, amyloid-related imaging abnormalities (ARIA), amyloid imaging, magnetic resonance imaging (MRI), prescribing information, accelerated approval, Food and Drug Administration

## Abstract

Lecanemab (Leqembi^®^) is approved in the United States for the treatment of Alzheimer’s disease (AD) to be initiated in early AD (mild cognitive impairment [MCI] due to AD or mild AD dementia) with confirmed brain amyloid pathology. Appropriate Use Recommendations (AURs) are intended to help guide the introduction of new therapies into real-world clinical practice. Community dwelling patients with AD differ from those participating in clinical trials. Administration of lecanemab at clinical trial sites by individuals experienced with monoclonal antibody therapy also differs from the community clinic-based administration of lecanemab. These AURs use clinical trial data as well as research and care information regarding AD to help clinicians administer lecanemab with optimal safety and opportunity for effectiveness. Safety and efficacy of lecanemab are known only for patients like those participating in the phase 2 and phase 3 lecanemab trials, and these AURs adhere closely to the inclusion and exclusion criteria of the trials. Adverse events may occur with lecanemab including amyloid related imaging abnormalities (ARIA) and infusion reactions. Monitoring guidelines for these events are detailed in this AUR. Most ARIA with lecanemab is asymptomatic, but a few cases are serious or, very rarely, fatal. Microhemorrhages and rare macrohemorrhages may occur in patients receiving lecanemab. Anticoagulation increases the risk of hemorrhage, and the AUR recommends that patients requiring anticoagulants not receive lecanemab until more data regarding this interaction are available. Patients who are apolipoprotein E ε4 (*APOE4*) gene carriers, especially *APOE4* homozygotes, are at higher risk for ARIA, and the AUR recommends *APOE* genotyping to better inform risk discussions with patients who are lecanemab candidates. Clinician and institutional preparedness are mandatory for use of lecanemab, and protocols for management of serious events should be developed and implemented. Communication between clinicians and therapy candidates or those on therapy is a key element of good clinical practice for the use of lecanemab. Patients and their care partners must understand the potential benefits, the potential harms, and the monitoring requirements for treatment with this agent. Culture-specific communication and building of trust between clinicians and patients are the foundation for successful use of lecanemab.

## Introduction

Lecanemab (Leqembi^®^), an anti-amyloid monoclonal antibody, is approved by the United States (US) Food and Drug Administration (FDA) for the treatment of Alzheimer’s disease (AD) initiated in early AD (mild cognitive impairment (MCI) or mild dementia due to AD). Treatment candidates should have amyloid pathology as demonstrated by amyloid positron emission tomography (PET) or cerebrospinal fluid (CSF) tests indicative of AD. Lecanemab was approved using the accelerated pathway available to the FDA for drugs that were developed to treat a serious or life threatening illnesses and have an effect on a biomarker considered reasonably likely to predict a clinical response, taking into account the prevalence of the condition and the availability or lack of alternative treatments (1). In the accelerated approval framework, a confirmatory trial is required to demonstrate clinical benefit and the agent can be withdrawn from the market if the confirmatory trial is negative.

In a Phase 2 trial, lecanemab was observed to produce marked lowering of amyloid plaques based on evidence derived from amyloid PET. Lecanemab has completed a confirmatory phase 3 trial (CLARITY-AD) and the FDA will review data from the CLARITY AD to determine if standard approval based on clinical outcomes of the trial is warranted (2). A decision by the FDAregarding standard approval of lecanemab is expected in mid-2023. The accelerated pathway was used recently for the approval of the anti-amyloid monoclonal antibody aducanumab (Aduhelm^®^) (3).

Marketed lecanemab (Leqembi^™^) is accompanied by Prescribing Information (PI) from the FDA that describes the indications and usage; dosage, forms, strengths, and administration; contraindications; warnings and precautions; adverse reactions; use in specific populations; development and preparation; clinical pharmacology; nonclinical toxicology; clinical studies; how supplied/storage and handling; and patient counselling information. The PI does not describe how to translate the information into clinical care, adjust clinical practices and workflow to use the agent safely, or present the potential benefits and harms to patients and care partners. Appropriate Use Recommendations (AURs) were developed to assist in guiding the use of new agents such as lecanemab into clinical practice and patient care and to anticipate challenges that may arise with use of a new therapy in real-world settings. Health care systems and advocacy groups may use the AURs to inform use of lecanemab. We describe the appropriate patient for treatment with lecanemab; dosing, administration, and monitoring of lecanemab; apolipoprotein E (*APOE*) genotyping of lecanemab treatment candidates; discussions with patients and care partners concerning lecanemab treatment; and clinician and workflow considerations regarding lecanemab use in practice. We describe AD-related populations for which there is no available information on the safe use of lecanemab.

The AUR for lecanemab does not discuss efficacy, reimbursement, or accelerated approval. The meaningfulness of the response to lecanemab has been debated, and we do not address that issue as part of use recommendations focusing on provision of lecanemab in community settings (4). The AUR is intended for use by clinicians who have decided to provide lecanemab as a treatment option for patients with early symptomatic AD. AURs are not intended to replace clinician judgment. Clinicians make management choices in the patient’s best interest in conjunction with patients and their care partners; these decisions may vary from the recommendations made in this AUR. All recommendations of the AUR are within the guidelines of the FDA-approved PI and fulfill the requirements of the on-label use of lecanemab. These recommendations may be adjusted as additional data are gathered.

## Method

This lecanemab AUR was developed by the Alzheimer’s Disease and Related Disorders Therapeutics Work Group (ADRD TWG) that was formed to address emerging issues in ADRD therapeutics. The ADRD TWG created the AUR for aducanumab (5). Collaborators with special expertise are invited to join TWG projects to augment the expert panel as occurred with the aducanumab AUR update and with the current lecanemab AUR (6). The AUR is based on the PI approved by the FDA, clinical trial data (Phase 2 and Phase 3), information from other drug treatments of dementia, review of the literature on AD and AD treatment, and expert opinion (2, 7–9). The AURs are based on all available data on lecanemab to date including the Phase 3 CLARITY AD trial.

### Lecanemab

Lecanemab is a recombinant humanized immunoglobulin gamma 1 (IgG1) anti-amyloid monoclonal antibody that binds to amyloid oligomers, protofibrils, and insoluble fibrils. Protofibrils represent a high molecular weight species of soluble amyloid and are preferentially bound by lecanemab (10). The murine version of lecanemab, mAb158, exhibits a binding preference for amyloid beta-protein (Aβ) protofibrils over Aβ monomers and a less marked binding preference for protofibrils over aggregated fibrillar amyloid. Lecanemab and mAb158 recognize soluble amyloid aggregates in human AD brain extracts (11, 12). The antibody is expressed in a Chinese hamster ovary cell line.

## Appropriate Patient for Treatment with Lecanemab

### Clinical Diagnosis

Lecanemab has been tested in patients with MCI due to AD and mild AD dementia diagnosed by the National Institute on Aging (NIA)-Alzheimer’s Association (AA) clinical criteria (13, 14) with confirmed amyloid pathology. To ensure that trial participants had mild severity of cognitive impairment, Mini-Mental State Examination (MMSE) (15) scores of 22–30 were required. Lecanemab’s efficacy and safety in less severe (e.g., preclinical AD (16)) and more advanced stages of AD dementia have not been established. We recommend that all patients being considered for lecanemab therapy meet diagnostic criteria for MCI due to AD or probable mild AD dementia with biomarker evidence (amyloid PET or CSF) of the AD pathophysiological process (Table 1).

### Clinical Features Relevant to Lecanemab

The lecanemab phase 2 and phase 3 studies excluded patients outside a specific age range (50–90 years old). Participants with immunologic disorders who were not adequately controlled or require therapy with immunoglobulins, systemic monoclonal antibodies, systemic immunosuppressants or plasmapheresis were excluded in the phase 3 CLARITY AD lecanemab study. Participants with other unstable medical conditions as well as those with stroke or transient ischemic attacks, bleeding disorders, or seizures in the previous 12 months were excluded in the phase 3 trial. Women who were pregnant or lactating were not be treated with lecanemab; no information is available on use in these situations. Individuals with depression who scored >8 on the Geriatric Depression Scale or had a body mass index (BMI) greater than 35 or less than 17 were excluded from both the phase 2 and 3 studies. We recommend adopting these same criteria while allowing medical judgment for individual circumstances when the impact of these modifications has been considered (Table 2). CLARITY AD excluded patients who had seizures within the past 12 months. A history of seizures may be related to symptoms of more severe amyloid related imaging abnormalities (ARIA; discussed in more detail below) including seizures and status epilepticus (6). We recommend excluding patients with any history of seizures until additional data are available. Cerebral amyloid angiopathy-related inflammation/amyloid beta-related angiitis (CAA-ri/ABRA) increase the risk for ARIA (discussed below) and should exclude patients as treatment candidates.

### Concomitant Medications

Lecanemab trials allowed the concurrent administration of symptomatic anti-dementia therapies (cholinesterase inhibitors and memantine). These agents can be allowed as concomitant medications by practitioners providing lecanemab. Patients receiving treatment for medical or psychiatric illnesses may be considered for treatment with lecanemab if the medical or psychiatric condition and the relevant medication doses are stable at the time of treatment initiation and if the patient is expected to be adherent to the schedule of infusions, scans, and clinical assessments required.

Aducanumab (Adulhelm^®^) is an anti-amyloid monoclonal antibody approved for the treatment of early AD. It is in the same class of agents as lecanemab and the treatment population is the same. The administration, titration, and magnetic resonance imaging (MRI) monitoring of aducanumab differ from those of lecanemab (6). Patients should not be given lecanemab if they are receiving aducanumab or had severe or recurrent ARIA with use of aducanumab.

Patients on anticoagulants are at higher risk for macrohemorrhage associated with lecanemab therapy. We recommend excluding patients from treatment with lecanemab if they are on warfarin, vitamin K antagonists, or direct oral anticoagulants (dabigatran, rivaroxaban, edoxaban, apixaban, betrixaban), or heparin until more evidence has accrued regarding the safety of administering lecanemab to patients on anticoagulants in the real-world practice setting. Severe, multi-focal brain hemorrhages leading to death were reported in a patient treated with tPA for acute stroke who had received lecanemab during an open-label extension (17). Lecanemab may increase the risk of hemorrhage from concomitant administration of thrombolytics (intravenous or intra-arterial), and we recommend that patients on lecanemab not be treated with acute thrombolytics until safety evidence of their combined use is available. Participants with clotting disorders should be excluded. Participants in lecanemab trials were allowed to continue or initiate treatment with aspirin. We recommend allowing patients on standard doses of aspirin (up to 325 mg/day) or other antiplatelet agents (clopidogrel, prasugrel, ticagrelor; at standard therapeutic doses) to be considered for treatment with lecanemab if they meet other criteria for therapy. Patients who are homozygous for the *APOE4* gene are at increased risk for ARIA with lecanemab administration, (see below) and the risk may be increased with antiplatelet agents.

### Imaging Recommendations

A positive amyloid biomarker --- either elevated amyloid on PET or elevated phosphorylated tau and low Aβ42 level (increased p-tau/ Aβ42 ratio) in the CSF --- is required prior to initiating treatment with lecanemab to establish that abnormal amyloid, the target of anti-amyloid MABs, is present. Positive tau PET is indicative of the presence of AD, but individuals may have a positive amyloid PET and a negative tau PET (18). Given this lack of concordance in some patients, we recommend use of amyloid PET to demonstrate the presence of the amyloid target when imaging is the biomarker modality used for confirmation. Reliable and approved blood biomarkers for AD pathology --- elevated plasma phospho-tau, decreased Aβ 42/40 ratio, abnormal profiles from combinations of biomarkers - may become fully validated soon but are not currently considered adequate to identify appropriate patients for treatment. High negative predictive values of some blood-based biomarkers suggests that negative results might be the basis for deciding which individuals are very unlikely to have AD and do not require further assessment (19).

A non-contrast MRI, utilizing TI fluid-attenuated inversion recovery (FLAIR) and T2*-weighted Gradient Recalled Echo (GRE) or equivalent sequences (such as susceptibility weighted imaging (SWI)), and diffusion-weighted imaging (DWI), preferably on a 3T magnet should be obtained to determine if an individual is a candidate for lecanemab therapy. If a pre-existing MRI with the aforementioned sequences obtained within 12 months prior to initiating therapy is available, it can be used to approve the patient for consideration for lecanemab therapy if abnormal amyloid status is confirmed and other recommendations are met. MRI-based exclusion criteria of the lecanemab phase 3 (CLARITY AD) study included a history of any CNS macrohemorrhage >10 mm in diameter, more than 4 microhemorrhages (<10 mm in diameter), evidence of superficial siderosis, evidence of brain vasogenic edema, significant white matter hyperintensities, multiple lacunar strokes, or any cerebral strokes involving a major vascular territory (20). Evidence of cerebral contusion, encephalomalacia, brain aneurysms or other vascular malformations, central nervous system (CNS) infection, and brain tumors other than meningioma or arachnoid cysts excluded patients from phase 3 trial participation. These same restrictions should apply when considering patients for treatment with lecanemab (Table 2). MRI evidence of underlying CAA-ri/ABRA or other conditions placing patients at risk for ARIA as well as more serious forms of ARIA should exclude patients as treatment candidates. Table 3 presents the criteria for probable CAA-ri with the corresponding MRI description (21).

Eligible patients must have a screening MRI within 12 months of treatment initiation to establish the potential safety of lecanemab therapy and MRI is also used to monitor for ARIA (discussed below). Those unable to undergo MRI due to claustrophobia, pacemaker, defibrillator, or metal implants are not eligible for lecanemab therapy. Computerized tomography (CT) is not adequate to detect the pathology that can render a patient inappropriate for lecanemab therapy or to monitor for ARIA once treatment is initiated.

## Appropriate Use of *APOE* Genotyping or Proteotyping When Considering Treatment with Lecanemab

The *APOE4* genotype represents the strongest genetic risk factor for sporadic AD (22–24). *APOE* in humans has three alleles: *APOE* ε3 (*APOE3*) is most common; *APOE* ε2 (*APOE2*) is least common and reduces the risk of AD; while *APOE* ε4 (*APOE4*), present in ~20–25% of the population, increases the risk of clinical AD in a dose-dependent manner. There is a significant interaction with sex, with female *APOE4* carriers at higher risk for AD than males, particularly at younger ages (25). *APOE4* genotype increases the risk of CAA and common AD co-pathologies such as cerebrovascular disease (22). Most patients with younger onset AD have at least one *APOE4* allele. In the CLARITY AD trial, 69% of participants had at least one allele; 53% were heterozygotes and 16% were homozygotes (2). The *APOE* gene produces the *APOE* protein, and some laboratory tests determine *APOE* status by assessing the patient’s proteotype.

*APOE4* carriers (especially homozygotes) are at increased risk for ARIA, symptomatic ARIA, and recurrent ARIA (discussed below; shown in Table 5). They are at increased risk for CAA-ri/ABRA, an additional risk factor for ARIA. We recommend *APOE* genotyping of all treatment candidates before initiating lecanemab therapy. This information will inform risk discussions and help guide safety considerations.

## Appropriate Dosing, Administration and Monitoring of Lecanemab

### Administration of Lecanemab

Lecanemab is administered intravenously every other week. No titration of lecanemab is required. Dosing is weight-adjusted, and patients are administered 10 milligrams per kilogram of body weight. Lecanemab is provided in vials of 500 mg/5 mL (100 mg/mL) or 200 mg/^2^ mL (100 mg/mL). It is added to an infusion bag containing 250 mL of 0.9% sodium chloride injection and administered through an intravenous line with a terminal low-protein binding 0.2 micron in-line filter. The infusion requires approximately 1 hour. As infusion reactions may occur (described below), patients should be observed for 3 hours after the first infusion with a follow-up telephone call later that day, as delayed infusion reactions are occasionally observed. The post-infusion observation period may be reduced to 2 hours for the second and third infusions and to 30 minutes for subsequent infusions if no infusion reactions have occurred. If an infusion is missed, administer the next dose as soon as possible and every two weeks thereafter.

The principal side effects of lecanemab are infusion reactions and ARIA. Post-treatment monitoring of patients is directed principally at detecting ARIA and guiding management decisions that minimize the likelihood of worsening or recurrence of ARIA. We recommend obtaining MRIs after the 5th, 7th, and 14th infusions as outlined in the PI. We recommend an additional week 52 (i.e., before the 26th infusion) MRI scan, especially for *APOE4* carriers and those with evidence of ARIA on earlier MRIs. This safety monitoring should be supplemented by unscheduled safety MRIs, obtained in response to the occurrence of symptoms potentially caused by ARIA. Symptoms of ARIA may be non-specific, such as headache and dizziness and may overlap with symptoms of AD, such as increasing confusion. Table 4 presents a list of the symptoms most commonly observed in patients with symptomatic ARIA. Deciding whether an unscheduled safety MRI is indicated requires clinical judgment based on the quality and intensity of the symptoms and the likelihood that they are produced by ARIA. Heightened vigilance for ARIA is warranted in *APOE4* carriers, especially homozygotes. After 12 months of treatment, obtaining MRIs should be guided by patient symptoms and prior MRI findings.

### Amyloid-Related Imaging Abnormalities

ARIA is a common side effect of treatment with amyloid-lowering monoclonal antibodies. Two types of ARIA occur: ARIA-E with edema and ARIA-H with hemorrhagic changes. ARIA is typically mild-moderate radiographically, asymptomatic, or mildly symptomatic clinically, and self-limited (reversible). ARIA occurred in 12.6% of all participants based on MRI in the CLARITY AD (phase 3) trial; 2.8% were symptomatic. Of the Phase 2 participants, 9.9% on the 10 mg/kg biweekly dose exhibited radiographic ARIA (i.e., the dose administered in the CLARITY AD phase 3 trial). Radiographic ARIA rates and the rates of ARIA with symptoms were substantially higher among patients with an *APOE* ε4 (*APOE4*) genotype, especially those homozygous for *APOE4*, compared to noncarriers (Table 5). In the CLARITY AD trial, the rate of ARIA in *APOE4* noncarriers was 5.4%, in *APOE4* heterozygotes it was 10.9%, and in *APOE4* homozygotes the rate was 32.6%. Rates of symptomatic ARIA were 1.4%, 1.7%, and 9.2%, respectively. These observations support *APOE* genotyping of all patients (who agree) to inform risk discussions with treatment candidates and their care partners (discussed in more detail below). Table 5 shows the rates of ARIA-E and ARIA-H for the Phase 2 and the Phase 3 (CLARITY AD) lecanemab trials (2, 9).

### ARIA Monitoring

**Figure 1.**
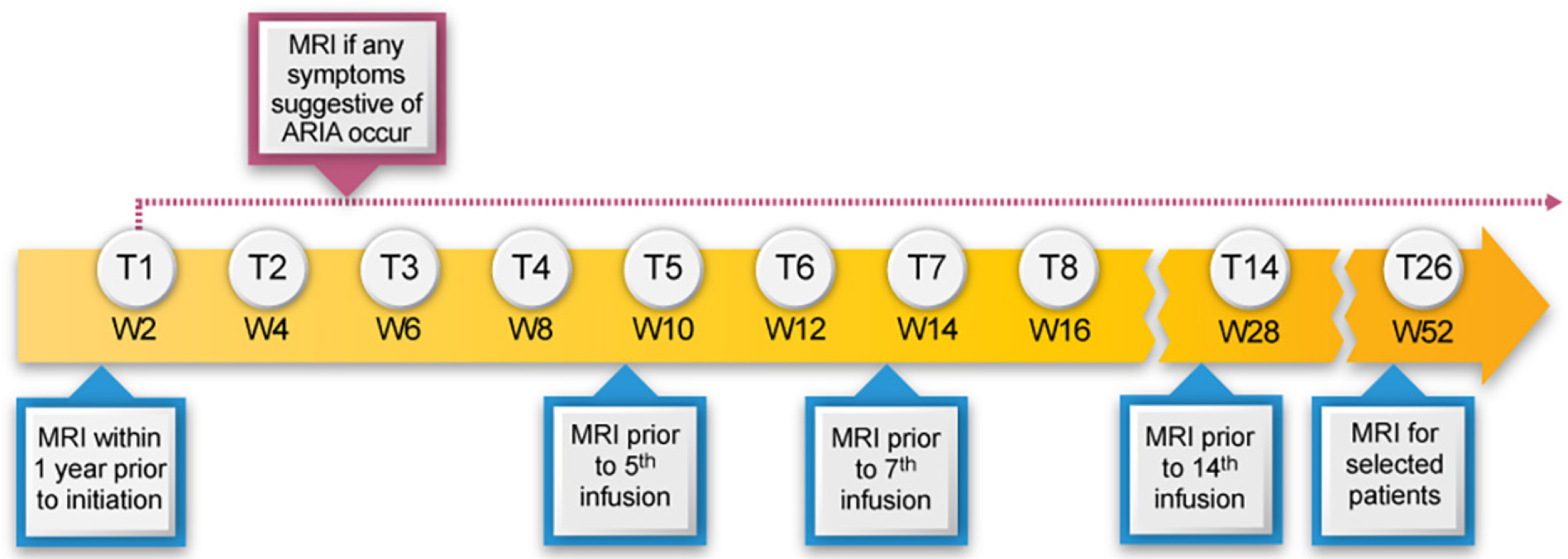
MRI monitoring for lecanemab

We recommend obtaining MRI scans prior to the 5th, 7th, and 14th infusions. We also suggest a week 52 (prior to the 26th infusion) MRI scan, especially in those who are *APOE4* carriers or had evidence of ARIA (whether with or without symptoms) on earlier MRIs (Figure 1). In CLARITY AD, ARIA-E tended to occur early, with most episodes (71%) detectable on the first or second MRIs obtained at weeks 9 and 13. Eighty-one percent resolved spontaneously within 4 months of radiographic detection. Initial episodes of ARIA-E continued to occur on safety MRIs at 24 and 52 weeks in *APOE4* carriers but were infrequent in non-carriers after week 13.

### Cerebral Macrohemorrhage

A cerebral macrohemorrhage represents a major central nervous system event often with enduring neurological deficits. A macrohemorrhage is more likely to occur when patients with pre-existing microbleeds or CAA-ri/ABRA are given anticoagulants (26, 27). Though numbers are small, the rate of cerebral macrohemorrhage in the CLARITY AD double blind and available open label periods was higher in patients on anticoagulation and in those on anticoagulation and lecanemab (Table 6). ARIA events with serious morbidity or mortality with lecanemab treatment are infrequent; three fatalities have been reported in the CLARITY AD open label extension which the site principal investigators attributed to lecanemab. One fatality occurred in an elderly *APOE4* gene non-carrier with cardiovascular disease on anticoagulation who developed a macrohemorrhage; a second death occurred in a patient homozygous for *APOE4* with a large vessel occlusion and pathologically confirmed severe CAA and vasculitis with multi-focal hemorrhage following tPA; a third death occurred in a patient homozygous for *APOE4* with severe ARIA-E and ARIA-H and a clinical syndrome resembling CAA-ri (16) (Table 6 (28)). We recommend that patients receiving anticoagulants not be treated with lecanemab (detailed above). If patients on lecanemab require treatment with anticoagulants, we recommend stopping the lecanemab infusions. Lecanemab therapy can be reinstated if anticoagulation is no longer medically indicated.

Taken together, *APOE4* gene carrier status, especially *APOE4* homozygosity, CAA-ri/ABRA, and anticoagulation are all risk factors for intracerebral bleeding. Caution should be used when considering lecanemab for patients who have these risk factors.

### Management of ARIA

**Figure 2.**
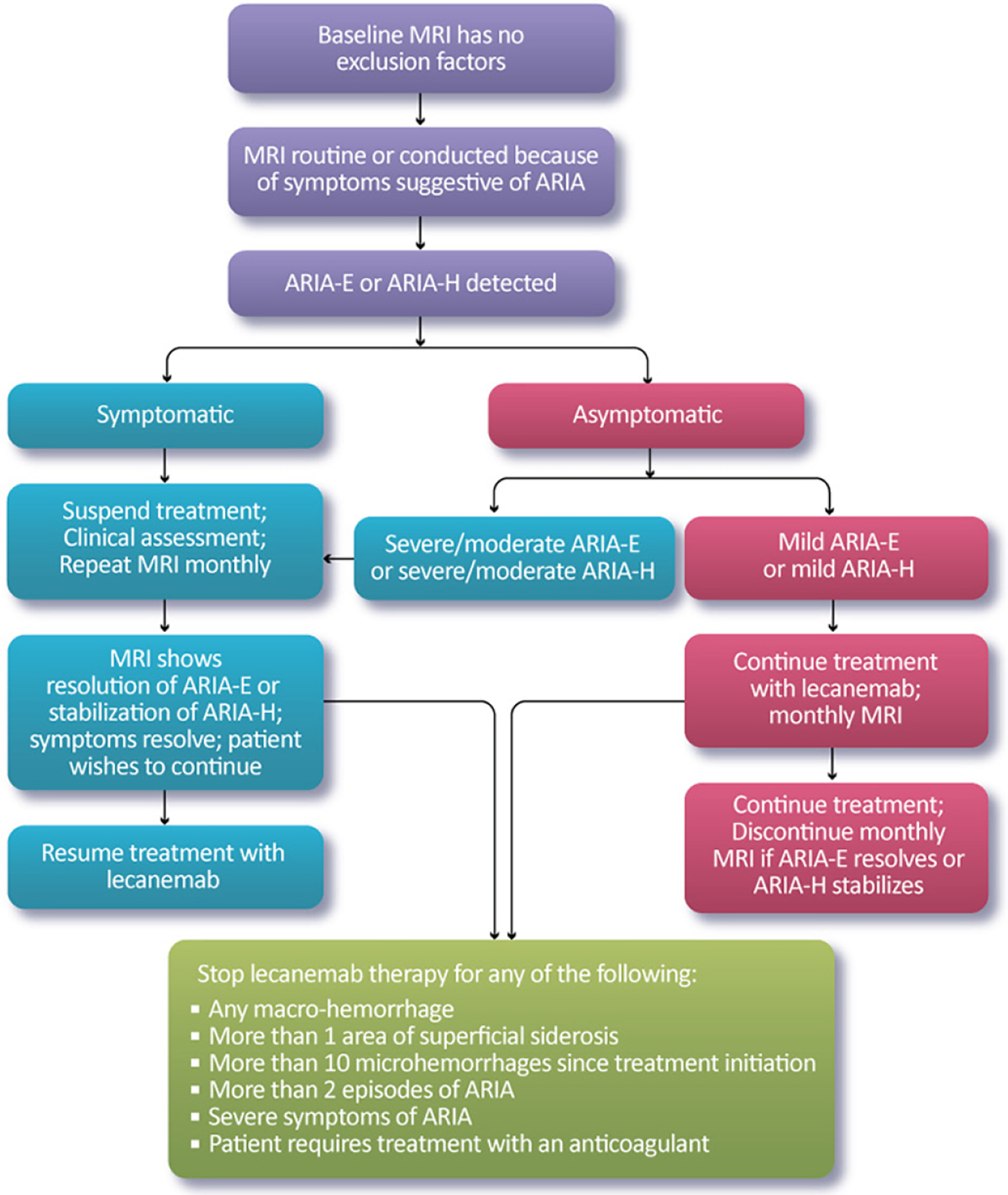
Monitoring and management of ARIA

Our recommendations for the management of ARIA with lecanemab are based on the ARIA management protocol in the CLARITY AD study and the management of ARIA with other amyloid-lowering antibodies (Figure 2). To detect ARIA early and mange it safely, radiologists and treating clinicians must recognize and interpret the pattern of MRI changes seen with ARIA-E and ARIA-H, know how to apply the ARIA management protocol in Figure 2, and recognize and manage the range of symptoms that may occur. Dosing can continue for individuals with radiographically mild asymptomatic ARIA recognized only on MRI (Table 7) with careful monitoring for symptoms and monthly non-contrast MRI scans until ARIA-E has resolved and ARIA-H has stabilized (Table 8). For those with moderate or severe radiographic ARIA or those with symptoms, lecanemab infusions should be suspended. Patients should receive careful clinical monitoring, management of symptoms, and monthly non-contrast MRI until ARIA-E resolves or ARIA-H stabilizes (Table 8). Once ARIA symptoms and radiographic ARIA-E changes resolve, restarting lecanemab can be considered based on a discussion of risks and benefits with the patient and family. Recurrent ARIA can occur. Decisions regarding ARIA management and when to discontinue treatment require consideration of the severity of the radiographic changes, presence and severity of any symptoms, patient’s *APOE* genotype, co-morbidities, and concurrent medications.

### Management of Serious and Severe ARIA

The decision to provide lecanemab therapy requires institutional preparedness for the management of serious and severe ARIA. Before patients are treated with lecanumab, a written protocol for the management of serious and severe ARIA should be developed, and the team of individuals possibly required for patient care should be informed about the lecanemab therapy program, the rare occurrence of severe or serious ARIA, and the availability of the written protocol. Table 9 lists the resources needed to ensure that the medical facility is prepared to manage patients with major ARIA consequences. Though rare, providers should be prepared to recognize and manage serious cases of ARIA, which resemble the syndrome of CAA-ri (29). Such cases typically manifest as large areas of edema with high signal on non-contrast FLAIR MRI sequences. Tissue swelling with mass effect from edema may be present and numerous microhemorrhages may be observed. *APOE4* carriers and especially *APOE4* homozygotes are at the highest risk for these events. No specific tests have been identified to predict which patients will experience this severe inflammatory form of ARIA. Patients may be asymptomatic, mildly symptomatic, or severely symptomatic. Seizures, status epilepticus, encephalopathy or stupor may occur, and lateralizing neurological signs may be observed. Severe ARIA may be reversible (30) or result in permanent disability or death (17, 31). When patients exhibit such severe symptoms and signs, an MRI scan should be obtained promptly to identify ARIA changes. The imaging should include DWI sequences to exclude an ischemic stroke. Admission to a hospital or critical care unit with personnel experienced in the management of cerebral edema may be needed. Early initiation of high-dose glucocorticoid treatment (e.g., methylprednisolone 1g intravenously per day for 5 days followed by an oral steroid taper over several weeks) should be considered. Monitoring for seizures and treatment, if they occur, should be part of the management protocol.

### Stopping Lecanemab for ARIA-Related Observations or Severe Infusion Reactions

We recommend discontinuing treatment with lecanemab for severe radiographic ARIA, severe symptomatic ARIA, any macrohemorrhage, one area of superficial siderosis, more than 10 microhemorrhages occurring since initiation of treatment, more than two episodes of ARIA or development of any medical condition that requires treatment with anticoagulants (Figure 2). We recommend considering discontinuation of lecanemab if patients have grade 3 or higher infusion reactions (discussed below; Table 10).

### Management of Infusion Reactions

Infusion reactions occurred in 26.4% of participants on lecanemab in the CLARITY AD study and were typically mild to moderate in severity. Infusion reactions usually occurred during the first 2 treatments and were seen during the infusion or up to several hours after the infusion. Infusion reaction symptoms typically resolve within 24 hours and can usually be managed at home. Symptoms include fever, chills, headache, rash, nausea, vomiting, abdominal discomfort, and elevated blood pressure. For more severe reactions (grade 2; Table 10 (32, 33)), the infusion should be stopped, and the patient treated with diphenhydramine and acetaminophen in milder cases and with oral dexamethasone (0.75 mg/day for 2–3 days) or oral methylprednisolone (80 mg twice per day for 2–3 days) when significant symptoms are present. The diphenhydramine or acetaminophen may be repeated every 4–6 hours until symptoms fully resolve. A call should be placed to the patient or care partner later that day to assess symptom resolution. For patients with grade 3 or higher infusion reactions (Table 10) the lecanemab infusion should be discontinued. Following an infusion reaction, patients should be pretreated with oral diphenhydramine 25–50 mg and oral acetaminophen 650 mg-1,000 mg 30 minutes prior to the next infusion. Low dose oral dexamethasone (0.75 mg 6 hours before infusion) or oral methylprednisolone (80 mg 6 hours prior to infusion) can be used for prophylaxis or treatment if diphenhydramine and acetaminophen are ineffective. Antihypertensive treatment may be needed for management of elevated blood pressure. Continue this prophylactic regimen until the patient remains asymptomatic in clinic and at home following 2–4 infusions. If a new infusion reaction occurs, oral diphenhydramine 25–50 mg and oral acetaminophen 650 mg-1,000 mg can be repeated every 4–6 hours post-infusion as needed with a contact later that day and the next day to determine if further treatment is needed. Although severe reactions are rare, clinics should be prepared with bronchodilators, oxygen, and epinephrine if needed.

The clinician can consider diphenhydramine or a topical corticosteroid cream for mild-moderate skin hypersensitivity reactions.

## Appropriate Discussions with Patients and Care Partners Concerning Treatment with Lecanemab

Patients with AD identified by their physicians as potential candidates for treatment with lecanemab must understand the potential benefits and potential harms of treatment. Care partners and family members of patients considering treatment with lecanemab must understand the benefits being sought, the occurrence of ARIA and its possible consequences, the possibility of infusion reactions, the need for twice monthly intravenous infusions, and the requirement for MRIs at baseline (if not done within the past 12 months) and 3 or 4 scans in the first year of therapy for the safe management of ARIA. Patients seeking treatment with lecanemab will be informed that *APOE* genotyping is recommended, there must be confirmation of amyloid pathology in the brain with either amyloid PET or CSF studies, and an MRI scan taken within the past 12 months must be available or obtained to ensure that they do not have vascular or other types of pathology incompatible with lecanemab therapy (described above). Patients with early AD have sufficiently preserved cognitive capacities to engage in and understand discussions regarding potential benefits, risks, and care requirements, yet they are challenged when there is substantial situational uncertainty (34). This must be considered in counseling patients about treatment with lecanemab. A variety of informational aids and decision-making tools have been identified and shown to be useful to assist the understanding of patients with cognitive impairment (35).

Treatment expectations for disease modifying therapies differ from those of symptomatic therapies. Cognitive enhancing agents such as the cholinesterase inhibitors and memantine temporarily improve function above baseline or temporarily postpone cognitive decline. They do not slow the rate of disease progression. The goal of disease-modifying therapies like lecanemab is slowing of the disease progression and cognitive decline; they are not expected to improve cognition or function.

The primary outcome of the phase 3 trial of lecanemab was the Clinical Dementia Rating-Sum of Boxes (CDR-SB) (36, 37). The CDR-SB is unfamiliar to patients and must be translated into terms that are more meaningful such as preserving cognition and function and remaining less dependent on others for longer periods of time. These are therapeutic goals that have been articulated as clinically meaningful by patients and their care partners (4, 38, 39). Potential slowing of loss of function, delayed progression from one global stage of AD to another, delay in disease milestones, delayed loss of quality of life for the patient, as well as reduced burden or loss of quality of life for the care partner may be more meaningful information than numerical CDR-SB scores (40).

*APOE* genotyping informs the risk of treatment with lecanemab (described above), and genotyping is recommended to inform discussions of lecanemab therapy. Genotyping of a treatment candidate that reveals the patient to be an *APOE4* gene carrier has implications for all first-degree relatives as they might might share the genetic risk. Counseling regarding genotyping and its ramifications has an important role in appropriate treatment discussions (41, 42).

In the phase 3 CLARITY AD trial, patients of Hispanic ethnicity comprised 12.5% and 12.3% of the lecanemab and placebo groups respectively. Asian patients represented 17.1% and 16.9% of the lecanemab and placebo participants. Black individuals represented only 2.3% and 2.7% of the lecanemab and placebo groups (2). There may be ethnic and racial differences in treatment responses, therapy preferences, adverse events, treatment adherence, and patient-care partner relationships. Differences in the frequency of amyloid positivity (e.g., less common in Asian, Hispanic, and Black compared to White individuals (43)) and differences in the relationship between *APOE4* and dementia between African Americans and White Americans (44) suggest that generalizations across ethnic and racial groups are not warranted. The modest number of racial and ethnic minority individuals included in the lecanemab trials provide limited insight into potential ethnic/racial differences in trial outcomes. This issue is particularly important in the US, where lack of diversity has been identified as a major challenge to research and treatment equity. Transparency concerning the small number of minority participants in lecanemab trials is important to acknowledge in discussions with minority individuals considering lecanemab therapy.

## Appropriate Adjustments and Workflow Considerations Regarding Lecanemab Use in Clinical Practice

Most clinicians have limited experience with lecanemab or other monoclonal antibody therapies for AD. These AURs are intended to help clinicians anticipate, plan, and implement the clinical practice and workflow alterations needed to accommodate lecanemab therapy. Clinician education regarding AD, cognitive assessment, amyloid confirmation with PET or CSF, ARIA detection and management, infusion and infusion reactions, *APOE* genotyping, and patient communication are foundational for success in lecanemab treatment programs. Table 11 describes the resources needed to implement and maintain a lecanemab treatment program. Development of a protocol for management of severe and serious ARIA is recommended for clinicians and institutions implementing a therapy program with lecanemab (described above; Table 9).

Clinicians should serially assess the mental status of all patients in their practice with MCI or dementia (45). Lecanemab is appropriate for patients with early AD like those included in the phase 2 and phase 3 clinical trials. Assessment of patients with evidence of cognitive impairment, complaints of cognitive decline, or observations from caregivers consistent with progressive cognitive impairment are triggers for determining if the patient is a candidate for further assessment and possible treatment (46).

Considering a patient for lecanemab therapy involves excluding non-AD causes of cognitive impairment, assessing a baseline or recent MRI for possible exclusionary factors, and confirming amyloid pathology with amyloid PET or CSF amyloid and tau assays (described above). Safe use of lecanemab requires periodic MRI imaging and urgent MRI studies if patients develop symptoms consistent with ARIA (described above). Clinical skills in assessing symptoms that may be indicative of ARIA and access to MRIs that can be made available rapidly are necessary for lecanemab treatment programs. Communication channels between patients and their caregivers and clinical staff must be established to allow rapid communication regarding symptoms suggestive of ARIA, infusion reactions, or other side effects. Guidance for patient counseling is available in the PI (www.accessdata.fda.gov).

Once treatment is initiated, supportive evidence of effectiveness may include less-than-expected decline on standard rating instruments performed by clinicians or change of trajectory of cognitive decline observed by family members (47). Confirmation of efficacy of lecanemab in the clinical setting is not feasible since the change in the rate of decline is relatively subtle, a change in rate of decline cannot be detected without multiple comprehensive trial-like assessments collected longitudinally, trajectories vary among and within patients, and comparison with a placebo group is not available.

Lecanemab is administered intravenously every other week and access to infusion centers, AD-specific infusion resources, in-office infusion settings, or home infusion opportunities is required. Infusion reactions are common with lecanemab administration; serious infusion reactions were uncommon (2, 9). Pre-infusion discussions with patients and care partners about the possibility of infusion reactions, institution of ameliorative therapy when reactions occur, and vigilance regarding possible serious reactions will be standard procedures for clinicians providing lecanemab therapy.

Communication with patients and their families regarding potential benefits and potential harms of treatment with lecanemab is essential for those administering this agent. Communication with informed professionals has become more urgent as social and electronic media have emerged as common sources of patient information, and misinformation found on the internet or purveyed through social media may influence patient decisions (48). Potential harms are balanced by discussions of potential treatment benefits of a disease-modifying agent like lecanemab and the implications of choosing no treatment (49). Communication that respects the cultural background of the therapy candidate is increasingly recognized as a key aspect of the patient-clinician information exchange. Lack of trust in a health care provider increases the likelihood of non-disclosure of health problems by the patient and could jeopardize appropriate management of lecanemab side effects (50). Comprehensive, transparent communication and building of trust between patients and clinicians will enhance the likelihood of successful and safe use of lecanemab in real-world clinical practice.

## Discussion

The goal of this AUR is to provide clinicians with information that facilitates the safe use of lecanemab in community practices. Patients participating in clinical trials differ from AD patients who are not included in trials. Trial participants are usually younger, healthier, better educated, have fewer comorbidities, and are less diverse than real-world patients. Use of lecanemab in older, less healthy, less well educated, and more diverse populations may result in efficacy and safety outcomes that differ from those observed in trials. Patients in trials are recruited and treated in expert centers and are closely monitored by expert clinicians with experience using lecanemab and other monoclonal antibodies. Trials use centralized readings of amyloid PET and MRI that are not available in community settings. These differences between providing lecanemab in a closely supervised clinical trial and providing this agent in community care practices indicate the need for vigilance to ensure the safe and effective use of lecanemab (51, 52). AURs are meant to anticipate the challenges to implementing lecanemab in practice. Post-marketing surveillance of putative side effects and the use of registries for patients receiving lecanemab or other monoclonal antibodies will facilitate data collection and guide any necessary use adjustments. We recommend that all patients receiving lecanemab be enrolled in the Alzheimer’s Network for Treatment and Diagnostics (ALZ-NET; www.alz-net.org) or similar registries as they become available. Education regarding use of lecanemab and other anti-amyloid monoclonal antibodies and emerging information regarding these agents will be available through ALZ-NET.

The efficacy and safety of lecanemab are known only for the type of patients that were included in clinical trials manifesting early AD with confirmed amyloid positivity (Tables 1 and 2). These AURs recommend patients be selected for initiation of treatment based on criteria like those of the completed lecanemab clinical trials; this is the population in whom efficacy and safety have been demonstrated.

The efficacy and safety of lecanemab for patients with AD dementia more severe than those included in the trials are not known, and recommendations for stopping lecanemab therapy when patients progress beyond mild AD dementia will depend on accruing more information. Key aspects of this discussion will depend on effectiveness observations collected by the clinician suggesting continuing disease slowing, safety observations regarding ARIA, and discussions with the patient and family members regarding the lack of trial information for this aspect of the illness and their impression of the potential benefits of continuing treatment.

Patients included in the lecanemab trials were in the early symptomatic stages of late age-of-onset AD, yet there are other populations of amyloid-bearing patients for whom treatment with anti-amyloid monoclonal antibodies may be useful. There is limited experience with early age-of-onset AD (EOAD), traditionally defined as symptom onset at age <65 years. Only 166 out of 859 lecanemab treated patients in CLARITY AD were 50 to 64 years of age at the time of enrollment. Such patients can be placed on lecanemab provided they meet the other treatment recommendations, but caution must be exercised as the clinical trials were not powered to provide safety or efficacy information in EOAD.

There are other populations of amyloid-bearing patients for whom treatment with anti-amyloid monoclonal antibodies may be useful. Patients with autosomal dominant AD (ADAD) were not specifically excluded from the lecanemab trials, and a small number may have been included. The specifics of safety and efficacy in these patients are unknown. Some ADAD-causing mutations are associated with a higher rate of CAA and patients with these CAA-related ADAD mutations should be excluded from current treatment with lecanemab. Information regarding which mutations are associated with CAA is available in the mutation database of Alzforum (www.alzforum.org). Clinical trials specifically designed for individuals with ADAD are available (53).

Persons with Down syndrome develop AOAD and are amyloid positive. There is an increased occurrence of CAA in patients with Down syndrome and they should be excluded from treatment with lecanemab (54). Clinical trials for patients with Down syndrome are under consideration and additional data including information that may guide the use of lecanemab in this population are expected (55).

Patients included in the lecanemab trials had relatively typical forms of memory-predominant AD. Patients with atypical AD syndromes including logopenic variant primary progressive aphasia, posterior cortical atrophy, or behavioral or dysexecutive AD have positive amyloid studies and may be candidates for lecanemab treatment. Patients with these syndromes were not specifically excluded from the lecanemab trials if they met the inclusion criteria for participation. The safety and efficacy of treatment in these patients has not been studied. The absence of treatment-related information for patients with atypical AD should be acknowledged in discussions with the therapy candidate and their care partner.

Individuals in the preclinical phase of AD who are cognitively normal and have positive amyloid PET or CSF studies were excluded from the completed lecanemab trials. An ongoing trial will determine if treatment with lecanemab in the preclinical phase of AD will prevent or delay the emergence of cognitive symptoms (56).

Excluding patients with some comorbidities and treatments including anticoagulants, severe cerebrovascular disease, and others may result in disproportionately excluding patients from underrepresented groups including Black/African Americans, Latinos, Asians, Native Americans and Pacific Islanders, and others with adverse determinants of health from being considered as candidates for lecanemab therapy. These individuals were under-represented in the lecanemab trials, but they may still qualify for lecanemab treatment if they meet the treatment criteria detailed herein. Transparent acknowledgement of the limited information available regarding treatment responses in these patients should be acknowledged in discussions with the patient and caregiver.

Spontaneous ARIA-like events occur in patients with CAA and ABRA (20, 29, 57). CAA/ABRA is associated with cerebral microbleeds which are a risk factor for ARIA (20, 29, 57, 58). Patients with underlying CAA-ri may be at particularly high risk for ARIA (59). Evolving information may improve our ability to identify patients who are at particularly high risk for ARIA events.

### Summary

This AUR presents information derived from the lecanemab clinical trials and from research studies and care experience with AD to assist practitioners who have chosen to provide patients with the option of treatment with lecanemab. We recommend that patients treated with lecanemab be like those included in lecanemab trials where safety and efficacy have been shown. Exclusion of patients with more than four microhemorrhages or other evidence of cerebrovascular disease is thought to reduce the risk of ARIA associated with lecanemab therapy. *APOE* genotyping is recommended to identify patients who are *APOE4* gene carriers, especially those homozygous for *APOE4* who are at higher risk for the occurrence of ARIA. We recommend that patients requiring treatment with anticoagulants not be given lecanemab and that thrombolytic therapy for ischemic stroke not be administered to lecanemab recipients. Lecanemab is given intravenously every other week and requires no titration. The AURs suggest an MRI be obtained at baseline and prior to the 5th, 7th, and 14th infusions and also after 52 weeks of therapy (for *APOE4* carriers or those with a history of ARIA) in order to monitor for asymptomatic radiographic ARIA. An MRI is obtained if patients have symptoms suggestive of an ARIA event. Infusion reactions are relatively common and may require prophylactic management with anti-inflammatory therapies. Thorough discussion of the expected therapeutic benefit of this disease- modifying therapy as well as the potential for harm are key elements in good clinical practice for the use of lecanemab.

## Figures and Tables

**Table 1.** Diagnostic criteria for mild cognitive impairment (MCI) due to AD and probable mild AD dementia with evidence of the AD pathophysiological process. Severity measures such as the Mini-Mental State Examination (MMSE) are used to define MCI and mild AD dementia (13, 14)

Syndrome	Definition
Mild cognitive impairment (MCI) due to AD (intermediate likelihood)	• Cognitive concerns by the patient, knowledgeable informant, or the physician • Objective impairment in one or more cognitive domains including memory, executive function, attention, language, and visuospatial skills. • Generally preserved activities of daily living • No dementia • Positive AD biomarker
Dementia	• Cognitive or behavioral impairment involving a minimum of two of the following domains: memory, executive function, visuospatial function, language, behavior • Cognitive impairment detected and diagnosed through a combination of (1) history-taking from the patient and a knowledgeable informant and (2) an objective cognitive assessment • Symptoms interfere with the ability to function at work or perform usual activities • Decline from previous levels of functioning • Symptoms not explained by delirium or major psychiatric disorder
Probable AD dementia with evidence of the AD pathophysiological process	• Meets criteria for dementia • History of worsening of cognition by report or observation • The initial and most prominent cognitive deficits are evident on history and examination in one of the following categories: - Amnestic presentation - Nonamnestic presentations associated with amyloid positive confirmation • Positive amyloid biomarker
Cognitive impairment severity	• MMSE score of 22–30 to define MCI and mild AD dementia

**Table 2.** Inclusion and exclusion criteria used in the CLARITY AD study and corresponding proposals of the Appropriate Use Recommendations (AUR)

Inclusion and Exclusion Criteria Applied in the Clarity AD Trial of Lecanemab	Appropriate Use Recommendations for Patients Considered for Treatment with Lecanemab
**Inclusion Criteria**	
Diagnosis of Mild Cognitive Impairment (MCI) or mild AD dementia	Clinical diagnosis of MCI or mild AD dementia as defined in Table 1
Objective impairment in episodic memory as indicated by at least 1 standard deviation below age-adjusted mean in the Wechsler Memory Scale IV-Logical Memory (subscale) II (WMS-IV LMII)	Clinical diagnosis of MCI or mild AD dementia as defined in Table 1
Positive biomarker for brain amyloid pathology	Positive amyloid PET or CSF studies indicative of AD
50–90 years of age	Physician judgement used for patients outside the 50–90 year age range
Mini Mental State Examination (MMSE) score > 22 at Screening and Baseline and < 30 at Screening and Baseline	MMSE 22–30 or other cognitive screening instrument with a score compatible with early AD
Body mass index (BMI) greater than (>)17 and less than (<) 35 at Screening	Physician judgement used for patients at the extremes of BMI
If receiving an acetylcholinesterase inhibitor (donepezil, rivastigmine, galantamine) or memantine or both must be on a stable dose for at least 12 weeks prior to Baseline	Patients may be on cognitive enhancing agents (donepezil, rivastigmine, galantamine, or memantine) for AD; patients may not be on aducanumab
Unless otherwise stated, participants must have been on stable doses of all other (that is, non-AD-related) permitted concomitant medications for at least 4 weeks prior to Baseline	Patients may be on standard of care for other medical illnesses (see below for specifics regarding anticoagulation)
Have an identified study partner	Have a care partner or family member(s) who can ensure that the patient has the support needed to be treated with lecanemab
Provide written informed consent	Patients, care partners, and appropriate family members should understand the requirements for lecanemab therapy and the potential benefit and potential harm of treatment
**Exclusion Criteria**	
Any neurological condition that may be contributing to cognitive impairment above and beyond that caused by the participanťs AD	Any medical, neurologic, or psychiatric condition that may be contributing to the cognitive impairment or any non-AD MCI or dementia
More than 4 microhemorrhages (defined as 10 millimeter [mm] or less at the greatest diameter); a single macrohemorrhage >10 mm at greatest diameter; an area of superficial siderosis; evidence of vasogenic edema; multiple lacunar infarcts or stroke involving a major vascular territory; severe small vessel; or other major intracranial pathology	More than 4 microhemorrhages (defined as 10 millimeter [mm] or less at the greatest diameter); a single macrohemorrhage >10 mm at greatest diameter; an area of superficial siderosis; evidence of vasogenic edema; more than 2 lacunar infarcts or stroke involving a major vascular territory; severe subcortical hyperintensities consistent with a Fazekas score of 3 (60); evidence of amyloid beta-related angiitis (ABRA); cerebral amyloid angiopathy-related inflammation (CAA-ri); or other major intracranial pathology that may cause cognitive impairment
Evidence of other clinically significant lesions on brain MRI at Screening that could indicate a dementia diagnosis other than AD	MRI evidence of a non-AD dementia
History of transient ischemic attacks (TIA), stroke, or seizures within 12 months of Screening	Recent history (within 12 months) of stroke or transient ischemic attacks or any history of seizures
Any psychiatric diagnosis or symptoms (example, hallucinations, major depression, or delusions) that could interfere with study procedures in the participant	Mental illness (e.g, psychosis) that interferes with comprehension of the requirements, potential benefit, and potential harms of treatment and are considered by the physician to render the patient unable to comply with management requirements
Geriatric Depression Scale (GDS) score > 8 at Screening	Major depression that will interfere with comprehension of the requirements, potential benefit, and potential harms of treatment; patients for whom disclosure of a positive biomarker may trigger suicidal ideation. Patients with less severe depression or whose depression resolves may be treatment candidates
Any immunological disease which is not adequately controlled, or which requires treatment with immunoglobulins, systemic monoclonal antibodies (or derivatives of monoclonal antibodies), systemic immunosuppressants, or plasmapheresis during the study	Any history of immunologic disease (e.g., lupus erythematosus, rheumatoid arthritis, Crohn’s disease) or systemic treatment with immunosuppressants, immunoglobulins, or monoclonal antibodies or their derivatives
Participants with a bleeding disorder that is not under adequate control (including a platelet count <50,000 or international normalized ratio [INR] >1.5 for participants who are not on anticoagulant treatment, example, warfarin)	Patients with a bleeding disorder that is not under adequate control (including a platelet count <50,000 or international normalized ratio [INR] >1.5 for participants who are not on anticoagulant)
Participants who are on anticoagulant therapy should have their anticoagulant status optimized and be on a stable dose for 4 weeks before Screening	Patients on anticoagulants (coumadin, dabigatran, edoxaban, rivaroxaban, apixaban, betrixaban, or heparin) should not receive lecanemab; tPA should not be administered to individuals on lecanemab
Any other medical conditions (example, cardiac, respiratory, gastrointestinal, renal disease) which are not stably and adequately controlled, or which could affect the participanťs safety or interfere with the study assessments	Unstable medical conditions that may affect or be affected by lecanemab therapy

AD - Alzheimer’s disease; CSF - cerebrospinal fluid; MCI - mild cognitive impairment; MRI - magnetic resonance imaging; PET - positron emission tomography

**Table 3.** MRI criteria for probable cerebral amyloid angiopathy-related inflammation (21)

**Probably CAA-ri**
Age ≥40 years of age
Presence of ≥1 of the following clinical features: headache, decrease in consciousness, behavioral change, or focal neurological signs and seizures; the presentation is not directly attributable to an acute ICH
**MRI shows unifocal or multifocal WMH lesions (corticosubcortical or deep) that are asymmetric and extend to the immediately subcortical white matter; the asymmetry is not due to past ICH**
Presence of ≥1 of the following cortico-subcortical hemorrhagic lesions: cerebral macrobleed, cerebral microbleed, or cortical superficial siderosis
Absence of neoplastic, infectious, or other cause

ICH - intracerebral hemorrhage; MRI - magnetic resonance imaging; WMH - white matter hyperintensity

**Table 4.** Symptoms observed in patients who develop symptomatic ARIA

• Headache • Confusion • Visual changes • Dizziness • Nausea • Gait difficulty • Serious ARIA o Seizures o Status epilepticus o Encephalopathy o Stupor o Focal neurological deficits

**Table 5.** ARIA rates reported for the Phase 3 (CLARITY AD) trial of lecanemab

	All Participants on Placebo (N = 897)	All Participants on Lecanemab (N = 898)	APOE4 Noncarrier : Placebo (N = 286)	APOE4 Noncarrier: Lecanemab (N = 278)	APOE4 Carrier: Placebo (N = 611)	APOE4 Carrier: Lecanemab (N = 620)	APOE4 Heterozygote: Placebo (N = 478)	APOE4 Heterozygote: Lecanemab (N = 479)	APOE4 Homozygote: Placebo (N = 133)	APOE4 Homozygote: Lecanemab (N = 141)
ARIA-E	1.7% (15/897)	12.6% (113/898)	0.3% (1/286)	5.4% (15/278)	2.3% (14/611)	15.8% (98/620)	1.9% (9/478)	10.9% (52/479)	3.8% (5/133)	32.6% (46/141)
Symptomatic ARIA-E	0	2.8% (25/898)	0	(1.4%) 4/278	0	3.4% (21/620)	0	1.7% (8/479)	0	9.2% (13/141)
Serious event with ARIA-E	0	0.8% (7/898)	0	0.7% (2/278)	0	0.8% (5/620)	0	0.4% ( 2/479)	0	2.1% (3/141)
Total ARIA-H (Concurrent & Isolated)	9.0% (81/897)	17.3% (155/898)	4.2% (12/286)	11.9% (33/278)	11.3% (69/611)	19.7% (122/620)	8.6% (41/478)	14.0% (67/479)	22.1% (28/133)	39.0% (55/141)
Symptomatic ARIA-H	0.2% (2/897)	0.7% (6/898)	0	0.4% (1/278)	0.3% (2/611)	0.8% (5/620)	0.2% (1/478)	1.0% (5/479)	0.8% (1/133)	0
Serious event with ARIA-H	0.1% (1/897)	0.6% (5/898)	0.3% (1/286)	0.7% (2/278)	0	0.5% (3/620)	0	0.2% (1/479)	0	1.4% (2/141)
Microhemorrhage	7.6% (68/897)	14.0% (126/898)	3.1% (9/286)	7.2% (20/278)	9.7% (59/611)	17.1% (106/620)	7.1% (34/478)	12.1% (58/479)	18.8% (25/133)	34.0% (48/141)
Superficial siderosis	2.3% (21/897)	5.6% (50/898)	0.7% (2/286)	4.7% (13/278)	3.1% (19/611)	6.0% (37/620)	2.7% (13/478)	4.0% (19/479)	4.5% (6/133)	12.8% (18/141)
ICH^2^ (Including non- TEAE)	0.2% (2/897)	0.7% (6/898)1	0.3% (1/286)	0.4% (1/278)	0.2% (1/611)1	0.8% (5/620)1	0.2% (1/478)1	0.6% (3/479)1	0	1.4% (2/141)
Isolated ARIA-H^3^	7.8% (70/897)	8.9% (80/898)	3.8% (11/286)	8.3% (23 / 278)	9.7% (59/611)	9.2% (57/620)	7.3% (35/478)	8.4% (40/479)	18.0% (24/133)	12.1% (17/141)
Symptomatic Isolated ARIA-H	0.2% (2/897)	0.4% (4/898)	0	0.4% (1/278)	0.3% (2/611)	0.5% (3/620)	0.2% (1/478)	0.6% (3/479)	0.8% (1/133)	0

1. Includes one non-treatment emergent adverse event (non-TEAE) case in each treatment group: event occurred during study but > 30 days after discontinuing study medication;

2. ICH - Intracerebral hemorrhage (>1cm) or macrohemorrhage;

3. ARIA-H in subjects who did not also experience ARIA-E at any time

**Table 6.** Cerebral macrohemorrhage in patients on lecanemab with data for those on anticoagulants (28)

Study	Total		On Anticoagulants	
	Placebo n/N(%)	Lecanemab n/N(%)	Placebo n/N(%)	Lecanemab n/N(%)
201 Double Blind Phase	0/245 (0%)	1/161 (0.6%)	0/20 (0%)	0/11 (0%)
201 Open Label Phase	NA	1/180 (0.6%)	NA	0/18 (0%)
301 Double Blind Phase	2/897 (0.2%)	6/898 (0.7%)	0/74 (0%)	2/83 (2.4%)
301 Double Blind Plus Open Label Phase	NA	10/1608 (0.6%)	NA	5/140 (3.6%)
301 Double Blind Plus Open Label Phase Deaths with Concurrent Macrohemorrhage	1/897 (0.1%)	2/1608 (0.1%)	0/74 (0%)	2/140 (1.4%)

NA – not applicable

**Table 7.** Description of mild, moderate, and severe radiographic ARIA (from the Prescribing Information)

	Radiographic Severity		
ARIA Type	Mild	Moderate	Severe
ARIA-E	FLAIR hyperintensity confined to sulcus and/or cortex/subcortex white matter in one location <5 cm	FLAIR hyperintensity 5 to 10 cm in single greatest dimension, or more than 1 site of involvement, each measuring <10 cm	FLAIR hyperintensity >10 cm with associated gyral swelling and sulcal effacement. One or more separate/independent sites of involvement may be noted
ARIA-H Microhemorrhage	≤ 4 new incident microhemorrhages	5 to 9 new incident microhemorrhages	10 or more new incident microhemorrhages
ARIA-H Superficial Siderosis	1 focal area of superficial siderosis	2 focal areas of superficial siderosis	> 2 areas of superficial siderosis

**Table 8.** Management of ARIA depending on the severity of symptoms and the severity of the radiographic ARIA-E or ARIA-H on MRI

	Symptom Description			
	No Symptoms	Mild Symptoms	Moderate Symptoms	Severe Symptoms
Severity of Changes Observed on MRI	None	Discomfort noted; no disruption of daily activity	Discomfort sufficient to reduce or affect normal daily activity	Incapacitating, with inability to work or to perform normal daily activity
**ARIA-E on MRI**				
Mild	Continue dosing	Suspend dosing	Suspend dosing	Discontinue dosing
Moderate	Suspend dosing	Suspend dosing	Suspend dosing	Discontinue dosing
Severe	Discontinue dosing	Discontinue dosing	Discontinue dosing	Discontinue dosing
**ARIA-H on MRI**				
Mild	Continue dosing	Suspend dosing	Suspend dosing	Discontinue dosing
Moderate	Suspend dosing	Suspend dosing	Suspend dosing	Discontinue dosing
Severe	Discontinue dosing	Discontinue dosing	Discontinue dosing	Discontinue dosing

**Table 9.** Medical Center resources needed to manage serious or severe ARIA

• Emergency department with resources to assess suspected or known ARIA
• MRI scanners readily available for unscheduled scanning of symptomatic patients
• Knowledgeable MRI readers proficient in detection and interpretation of ARIA
• Clinicians with experience in the management of cerebral edema or ARIA
• Hospital ward for monitoring and management
• Intensive care unit availability
• Electroencephalography available to inpatients
• Neurologist with experience in management of seizures and status epilepticus

**Table 10.** Grading of infusion reactions (32, 33)

Grade 1	Grade 2	Grade 3	Grade 4	Grade 5
Mild transient reaction; infusion interruption not indicated; intervention not indicated	Infusion interruption but responds promptly to symptomatic treatment (e.g., antihistamines, acetaminophen, NSAIDs, narcotics, i.v. fluids); prophylactic medication indicated for < 24 hours	Prolonged recurrence of symptoms following initial improvement; hospitalization may be indicated for clinical sequelae (e.g., poorly controlled hypertension)	Life-threatening consequences; urgent intervention indicated (may require pressor or ventilatory support)	Death

**Table 11.** Resources needed by a clinician or medical center for the safe and effective use of lecanemab

• Clinician skilled in the assessment of cognition to identify individuals with mild cognitive impairment or mild dementia due to Alzheimer’s disease
• MRI available for baseline assessment of cerebrovascular pathology and for monitoring of amyloid related imaging abnormalities (ARIA)
• Radiologists, neurologists, or other clinicians expert in the identification and interpretation of cerebrovascular lesions and ARIA
• Amyloid positron emission tomography or lumbar puncture capability to determine the amyloid status of treatment candidates
• Radiologists, nuclear medicine specialists, neurologists, or other specialists skilled in the interpretation of amyloid imaging or neurologist, radiologists, or other clinicians skilled in the conduct of lumbar puncture
• Apolipoprotein E genotyping resources
• Genetic expertise to counsel patients on the implications of apolipoprotein E genotyping
• Expertise in communicating with patients and care partners regarding anticipated benefits, potential harm, and requirements for administration and monitoring while on lecanemab
• Infusion settings that can be made available every two weeks to patients receiving therapy
• Knowledgeable staff at infusion sites capable of recognizing and managing infusion reactions
• Communication channels established between experts interpreting MRIs and clinicians treating patients with lecanemab
• Communication channels established between clinicians treating patients with lecanemab and the patient and care partner
• Availability of hospital resources including intensive care unit
• Expertise in the management of seizures and status epilepticus for patients with severe or serious ARIA
• Protocol with standard operating procedures for management of serious and severe ARIA
